# Communication spectrum prediction method based on convolutional gated recurrent unit network

**DOI:** 10.1038/s41598-024-56311-y

**Published:** 2024-04-18

**Authors:** Lige Yuan, Lulu Nie, Yangzhou Hao

**Affiliations:** 1 Information Engineering College, Zhengzhou Technology and Business University, Zhengzhou, 451400 China; 2 Underground Pipeline Detection Institute, Mineral Resources Exploration Center of Henan Geological Bureau, Zhengzhou, 450053 China

**Keywords:** Recurrent neural network, Convolutional, Door control unit, Communication spectrum, Prediction, Radio, Computer science, Information technology

## Abstract

In modern wireless communication systems, the scarcity of spectrum resources poses challenges to the performance and efficiency of the system. Spectrum prediction technology can help systems better plan and schedule resources to respond to the dynamic changes in spectrum. Dynamic change in the spectrum refers to the changes in the radio spectrum in a wireless communication system. It means that the available spectrum resources may change at different times and locations. In response to this current situation, this study first constructs a communication collaborative spectrum sensing model using channel aliasing dense connection networks. Then, combining convolutional neural network and gated cyclic unit network in deep learning technology, a communication spectrum prediction model is built. It aims to achieve accurate perception and prediction of spectrum resources through the aforementioned spectrum sensing and prediction models. The results confirm that the proposed perception model has inconsistent perception accuracy under different number of secondary users, with a maximum of 0.99. It is verified that the proposed spectrum prediction model achieves a high prediction accuracy of 0.95 within 208 s and its performance outperforms current similar models. The results are based on the model's deep learning analysis of massive historical communication data, in which the optimized shuffle dense net model plus convolutional gated recurrent unit model is the key to achieve fast and accurate prediction. On the contrary, the highest spectrum prediction accuracy of Recurrent Neural Network (RNN), Long Short-Term Memory (LSTM), and Convolutional Neural Networks-Long Short-Term Memory (ConvLSTM) models are 0.86, 0.90, and 0.85, respectively. And the model needs to run for a longer period of time, up to 324, for ConvLSTM to reach the prediction accuracy value of 0.95. In summary, the perception and prediction model built by this research has good performance, and its application in the field of wireless communication can assist staff in better monitoring spectral changes, thereby making more efficient use of spectral resources.

## Introduction

With the rapid development of wireless communication technology, the efficient management of spectrum resources has become a crucial issue^1^. Especially in advanced communication standards such as Long-Term Evolution (LTE) and 5th Generation (5G), the demand for spectrum resources has increased dramatically. Effective spectrum management not only requires real-time and accurate spectrum usage sensing, but also requires prediction of future spectrum usage in order to optimize resource allocation and network planning^2,3^. In current research, spectrum sensing technology and spectrum prediction technology have been widely used. Spectrum sensing technology identifies and analyzes the spectrum energy observation matrix to achieve accurate perception of spectrum usage^4,5^. At the same time, spectrum prediction technology improves the ability to accurately predict the status of communication spectrum through long-term prediction on multiple time slots. However, existing spectrum sensing methods and prediction models still have some limitations in sensing accuracy, prediction speed, and long-term dependency processing^6^. Based on the above research background, this study innovatively optimizes the existing spectrum sensing and prediction techniques. Firstly, focusing on the spectrum sensing, a model for communication collaboration based on channel-mixed densely connected networks is proposed. The model aims to accurately capture and analyze the current spectrum usage in real-time, providing the necessary basic data and prerequisites for spectrum prediction. Subsequently, the study further proposes a spectrum prediction model based on Convolutional Neural Network (CNN) and gated recurrent unit networks, which will further predict future spectrum usage trends and patterns on the basis of the perception model. The combination of the spectrum perception model and the spectrum prediction model can provide a comprehensive spectrum management solution for wireless communication systems. The perception model provides accurate real-time spectrum usage data, while the prediction model uses these data to predict future spectrum demand, thus enabling wireless network operators to plan and utilize spectrum resources more efficiently.

This study consists of five parts in total. Firstly, there is an introduction to the entire article. Secondly, it is a summary and analysis of the related research. The third part specifically introduces how to build the spectrum perception model and the spectrum prediction model. Next is the performance verification of these two models. Finally, there is a summary of the research results and an analysis of future research directions.

## Related works

CNN and Long Short-Term Memory (LSTM) are widely known methods in deep learning, especially in computer vision, which have been widely applied and improved. Li et al. found that the process of synthetic-aperture radar and laser imaging was often accompanied by multiplicative noise. To solve this problem, researchers use the alternating direction multiplier method based on depth CNN to denoise Apriori algorithm. This method can achieve better image visual effects^7^. Wu et al. noticed that the random placement of parts during the manufacturing process made it difficult for robots to recognize and operate. Therefore, researchers proposed a cascaded CNN robot grasping method that utilized monocular vision and small datasets of dispersed parts. This method achieves part grasping through a robotic arm with a camera and a learning method, where CNN is used to extract key points of scattered parts, improving success rate. This method can effectively assist robots in recognition and grasping tasks^8^. Shalash et al. proposed a system that could estimate driver fatigue status using only one Electroencephalography (EEG) signal channel to improve estimation efficiency. The system converts the received black and white EEG into color images, and then uses CNN to identify the fatigue status of drivers. The researchers identified the three most accurate EEG signal channels and verified through experiments that the system's estimation accuracy for these three channels was 94.33%, 92.57%, and 93%, respectively^9^. To solve the problem of insufficient accuracy of existing ship trajectory prediction models, Liang et al. proposed an LSTM ship trajectory prediction model based on adaptive particle swarm optimization. The model optimizes and improves the parameters of LSTM through particle swarm optimization, including the number of hidden layer nodes, learning rate, maximum iteration, and input layer step size. Compared with other models, this prediction model has higher accuracy^10^. Peng et al. pointed out that existing software crowdsourcing recommendation mechanisms did not consider contextual information of crowdsourcing tasks. And they proposed a new LSTM recommendation framework for worker capability correction. By integrating LSTM to extract long-run and short-run feature layers and attention mechanisms, the framework can accurately assess changes in the interests and preferences of crowdsourcing workers in historical tasks. So it can effectively improve the quality and efficiency of crowdsourcing recommendations^11^.

Ding et al. conducted research on spectrum sharing satellite systems and proposed a Spectral Prediction Model (SPM) based on Convolutional Neural Network-Bidirectional Long Short-Term Memory (CNN-BiLSTM) neural network. The historical spectrum occupation data of geosynchronous orbit are preprocessed and predicted using the CNN-BiLSTM neural network model. This prediction model has good performance, higher prediction accuracy than traditional CNN and LSTM models, and lower average error values^12^. Bhowmik and Malathi proposed a hybrid prediction model to improve the spectral efficiency of Cognitive Radio (CR). This model consists of an actor critical neural network optimized based on krill-herd whale and a hidden Markov model. The model has better throughput performance, with a maximum sensing time of only 650 s^13^. Silva et al. optimized the spectral efficiency in cellular networks, and they studied two modes: full duplex and dynamic time division duplex. These two optimized duplex modes can suppress cross link interference and improve the capacity and user throughput of cellular networks^14^. Ren et al. utilized a cooperative spectrum acquisition model to improve the spectrum acquisition rate and alleviate channel interference issues in large-scale wireless networks. In addition, they also introduced spectrum access level and user participation level as new performance testing indicators. The cooperative spectrum acquisition model performs well in terms of spectrum access level and user participation level and improves the spectrum acquisition efficiency of wireless network systems^15^. In direct sequence spread spectrum communication systems, the traditional dual differential phase shift keying modulation does not perform well in time-varying environments because this modulation method amplifies phase noise. To address this problem, Sun D et al. proposed an iterative receiving method, which is suitable for time-varying underwater acoustic channel spectrum. The method first combines interleaved coding modulation with multi-symbol differential detection and iterative decoding. Doppler shift is used to track channel phase changes through a dynamic linear prediction model. Finally, an adaptive reference signal selection algorithm is introduced to increase the amplitude of the correlation peak. Experimental results show that under constant acceleration conditions, this method achieves a gain of about 9 dB compared with the traditional dual differential phase shift keying modulation. And this method can also achieve 96 frames of error-free communication in deep-sea experiments^16^. To address the challenges of intelligent spectrum sensing, Yang et al. proposed a federated spectrum learning framework. The framework consists of a federated learning algorithm combined with reconfigurable smart surfaces. By deploying a well-trained CNN on each reconfigurable smart surface controller, the framework's perception and analysis capabilities for smart spectrum are effectively improved. Experimental results show that as the number of reconfigurable smart surface controllers and reflective elements increases, the federated spectrum learning framework can achieve higher spectrum prediction accuracy^17^. Orthogonal frequency division multiplexing has gradually developed into a popular modulation scheme in wireless communication systems and is widely used in technologies such as LTE and 5G. In wireless communication systems, the nonlinear power spectrum produced by radio frequency amplifiers can cause distortion to the channel and adjacent channels, thereby reducing transmission quality. Yan et al. proposed a power spectrum prediction model using Taylor polynomials, aiming to accurately predict the power spectrum of the orthogonal frequency division multiplexing basic signal caused by the nonlinearity of the radio frequency amplifier. Research results show that the proposed model can accurately predict the power spectrum to avoid signal distortion during transmission^18^.

Based on the above related studies, deep learning methods have been widely used in the image denoising, robot grasping, EEG signal analysis, ship trajectory prediction, and software crowdsourcing recommendation. They are chosen to predict the communication spectrum based on the following key reasons. First, they can effectively handle the high complexity and nonlinear characteristics of communication spectrum data. Second, they excel in handling high-dimensional data and real-time dynamic adjustment, enabling them to adapt to rapid changes in spectrum usage. Finally, their successful application in image processing, signal analysis, and other related fields provides empirical support for their effectiveness in communication spectrum prediction. X. Wang et al. proposed a spectrum prediction method based on a backpropagation-LSTM model to accurately predict future spectrum trends and channel states to optimise spectrum utilisation for CR technologies. The effectiveness of the improved model in handling time series is demonstrated by comparing it with a conventional neural network model. Simulation results show that the model performs better in terms of prediction performance and the time-series length has a significant impact on the prediction accuracy of the deep learning model^19^. Mishra and Chaudhary proposed a deep learning-based data driven model to automatically classify the received raw signal data. In the paper, various deep learning models are compared and analyzed. The performance comparison is done with different sample lengths and Signal to Noise Ratio (SNR) values and the results show that the ResNet model exhibits the highest detection probability at both low and high SNR. The method performs well in reducing the loss due to false alarms and increasing the spectrum detection probability, enabling better spectrum sensing^20^. Zhao proposed a novel temporal spectral ambient map prediction method aimed at solving the challenge of spectral ambient prediction in highly dynamic spectral environments. The method is based on radiated source knowledge and optical flow driven by a propagation channel model. First, an innovative radiated source localisation strategy is designed to obtain radiated source movement information. Then, the optical flow field of the available spectral environment map is combined with the radiation source movement information. Finally, a propagation model-driven reconstruction technique is developed to predict future temporal spectral environment maps. Simulation results show that this method is effective in capturing the spatio-temporal correlation of temporal spectral ambient maps and outperforms existing techniques in single- and multi-step temporal spectral ambient map prediction^21^.

In conclusion, although deep learning methods such as CNN and LSTM have shown significant application potential in several fields, especially in the radio spectrum prediction, there are still some key gaps in existing research. First, most research has focused on single applications or specific aspects of problem solving and lacks a comprehensive and systematic approach to spectrum management. For example, while denoising algorithms for multiplicative noise and methods to improve the success rate of robot grasping exist, they usually fail to comprehensively consider the overall optimization of radio spectrum management. Second, while deep learning models excel at processing complex data, the integration and coordinated use of these models for spectrum prediction and sensing is not yet mature enough. For example, Li et al. emphasized the effectiveness of deep convolutional networks in removing multiplicative noise, while Wu X et al. demonstrated the application of CNN in improving the success rate of robotic grasping tasks. Similarly, Shalash W M et al. utilized CNN to improve the accuracy of EEG signal analysis. Most of the existing studies focus on a single application of the model, such as image processing or EEG signal analysis, rather than its application in the broader context of spectrum management for wireless communications. In addition, existing studies often lack efficiency and flexibility in the allocation and management of spectrum resources. As a limited and valuable resource, the optimal allocation and use of spectrum resources is crucial for improving the overall performance of wireless communication systems. Based on this current research, the motivation of this study is to develop a more comprehensive and coordinated spectrum management scheme. By incorporating state-of-the-art deep learning techniques, this study proposes a collaborative spectrum sensing model for communication based on channel-mixed densely connected networks, and a spectrum prediction model combining CNN and gated recurrent unit networks. This integrated research approach aims not only to improve spectrum utilization efficiency, but also to optimize the allocation and management of spectrum resources. This new model is expected to provide more efficient and smarter spectrum management solutions for future wireless communications, thus promoting the further development of wireless communications technology.

## The spectrum sensing technology and spectrum prediction technology for wireless communications

**Figure 1:**
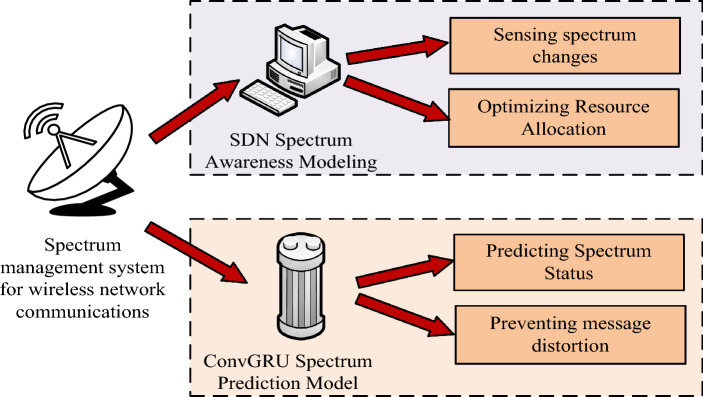
Overall research framework diagram.

In the rapidly developing field of wireless communications, efficient management of spectrum resources has become a crucial issue. This study conducts an in-depth discussion on spectrum sensing and prediction technology and proposes two innovative model designs: a communication cooperative spectrum sensing model based on channel aliasing dense connection network, and a convolutional gated recurrent neural network based on communication spectrum state prediction model. The overall framework structure diagram of the entire research is shown in Fig. 1.

Figure 1 shows the overall framework of this research. This research is mainly divided into two parts, namely building a spectrum sensing model and a spectrum prediction model. These two models are proposed to accurately monitor and predict spectrum usage status to improve the overall utilization efficiency of spectrum resources in wireless communication systems.

### Design of spectrum sensing model for communication cooperation based on channel aliasing dense connection network

Spectrum sensing technology refers to sensing and monitoring the usage of radio spectrum in wireless communication systems in order to effectively utilize spectrum resources. Spectrum sensing technology can detect, identify, and monitor idle or underutilized frequency bands in the radio spectrum in real time so that other wireless devices can communicate on these idle frequency bands, thereby improving the operating efficiency of the entire communication system.

**Figure 2:**
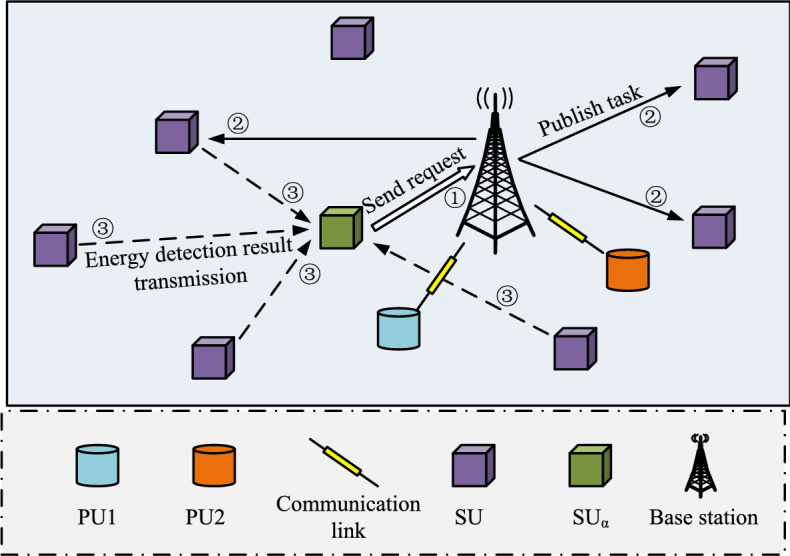
Spatial distribution map of the CR-NOMA spectral perception model.

CR is a wireless communication technology. Its core idea is to enable wireless devices to have intelligent cognitive and adaptive capabilities and be able to autonomously perceive, analyze and make decisions on spectrum usage. Non-Orthogonal Multiple Access (NOMA) is also a wireless communication technology used to enable multiple users to communicate simultaneously on the same frequency band^22^. Traditional wireless communication systems usually use static spectrum allocation, which allocates fixed spectrum resources to each wireless device. However, most spectrum resources are not fully utilized at different times and locations, resulting in a waste of spectrum resources. Based on this background, this research combines CR and NOMA, using CR technology to give the communication system intelligent sensing and decision-making capabilities to more effectively utilize spectrum resources. NOMA technology allows multiple users to communicate on the same site. Non-orthogonal data are transferred on the frequency band. The advantage of Cognitive Radio-Non-Orthogonal Multiple Access (CR-NOMA) technology is that it improves spectrum utilization and system capacity through intelligent sensing and allocation of spectrum resources. At the same time, through the NOMA technology, multiple users can perform parallel data transmission on the same frequency band, providing higher system throughput and user experience. Figure 2 shows the CR-NOMA spectrum sensing model.

The spatial distribution of the spectrum-aware model under CR-NOMA technique is shown in Fig. 2. In Fig. 2, Primary User (PU) and Secondary User (SU) are not only randomly distributed spatially through different shapes, but also perceive spectrum occupancy through energy detection. Specifically by analyzing the signal strength received by the SU to distinguish the active state (transmitting) and idle state (not transmitting) of the PU to accurately determine the spectrum occupancy. PU1 and PU2 denote two different PUs, respectively. ${\text{SU}}_{{\text{a}}}$ represents the cluster heads. In addition, it is shown in Fig. 2 how to divide the spatial location of the entire enclosed area into an equal area grid and ensure that the location of each SU is in the grid. NOMA is used to connect multiple PUs to the same frequency band and ensure that the transmit power of each PU is constant. 0 and 1 indicate that the PU is currently in idle and operational states, respectively. The spatial location of the entire closed area is divided into $P \times Q$ equal area networks and ensures that the location $\left( {x_{n} ,y_{n} } \right)$ of each SU is in the grid. All SUs in space use energy detection to perceive the energy information emitted by PU in the current task frequency band, treating SU as a receiver and PU as a transmitter.

The $t$-th sampled signal received by the $n$-th SU in the perception gap can be calculated using Eq. (1). Equation (1) is expressed as follows.

1 $$y_{n} \left( t \right) = \sum\limits_{m = 1}^{M} {s_{m} } \sqrt {\Omega_{m} } h_{m,n} x_{m} \left( t \right) + \delta_{n} \left( t \right)$$

where $s_{m}$ represents the working state of the $m$-th PU at the current moment $t$, $\Omega_{m}$ represents the transmission power of the $m$-th PU in its current operating state, $h_{m,n}$ represents the channel gain between the $m$-th PU and the $n$-th SU, $x_{m} \left( t \right)$ represents the transmission signal of the $m$-th PU, and $\delta_{n} \left( t \right)$ represents the Gaussian white noise of the $n$-th SU.

The relationship between PU, SU and channel gain can be expressed by Eq. (2), which is shown below.

2 $$h_{m,n} = k \times d^{ - a} = k \times \sqrt {\left( {x_{m} - x_{n} } \right)^{2} + \left( {y_{m} - y_{n} } \right)^{2} }^{ - a}$$

where $k$ represents fixed transmission loss, $d$ represents the distance between the transmitter and receiver, $\left( {x_{m} ,y_{m} } \right)$ and $\left( {x_{n} ,y_{n} } \right)$ represent the spatial coordinates of the $m$-th PU and the $n$-th SU, respectively, and $a$ represents the path loss index.

From Eq. (2), when the distance between the receiver and the transmitter is farther, the spectrum signal perceived by the SU is weaker. Based on Eq. (2), the formula for the observed value of spectral energy in the sensing gap can be further obtained as shown in Eq. (3).

3 $$e_{n} = \sum\limits_{t = 1}^{T} {\left| {y_{n} \left( t \right)} \right|}^{2}$$

where $T$ represents the number of signal samples at the target frequency point, $e_{n}$ represents the observed value of spectral energy, and $\left| {y_{n} \left( t \right)} \right|$ represents the modulo operation on the spatial ordinate of the $n$-th SU at time $t$.

Dividing the $P \times Q$ equal-area network based on spatial location, the cluster head will express the energy observations of each SU combined with the location information in the form of a matrix. Equation (4) is utilized to represent the matrix, which is expressed as follows.

4 $$E_{PQ} = \left[ {\begin{array}{*{20}c} {e_{11} } & {e_{12} } & \cdots & {e_{1Q} } \\ {e_{21} } & {e_{22} } & \cdots & {e_{2Q} } \\ \vdots & \vdots & \ddots & \vdots \\ {e_{P1} } & {e_{P2} } & \cdots & {e_{PQ} } \\ \end{array} } \right]$$

where $E_{PQ}$ represents the spatial spectrum energy observation matrix and $e_{PQ}$ represents the energy observation values possessed by each grid in the matrix.

The exact value of $e_{PQ}$ is calculated using Eq. (5), which is shown below.

5 $$e_{PQ} = \left\{ {\begin{array}{*{20}l} 0 \\ {e_{n} } \\ \end{array} } \right.$$

where there is no SU in grid $P \times Q$, $e_{PQ} = 0$, and on the contrary $e_{PQ} = e_{n}$.

Through Eqs. (1) to (5), the gray scale map of the perceived energy observation matrix in a certain space can be obtained, so that the positional change of SU and PU as well as the working status can be concluded.

**Figure 3:**
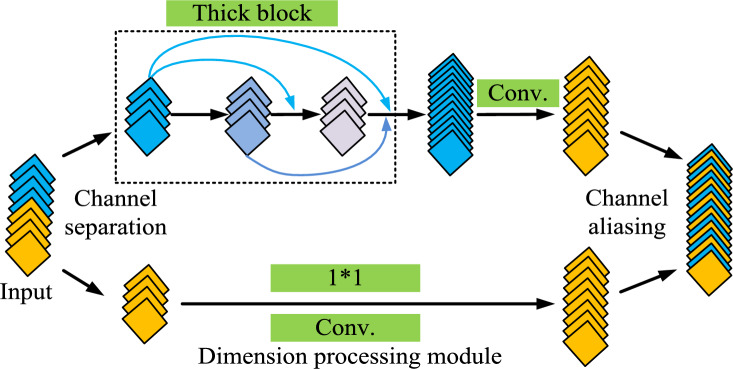
Structural diagram of SDB.

To further identify the grayscale map of the sensed energy observation matrix, the study proposes a communication collaborative spectrum sensed energy identification model using Shuffle Dense Network (SDN), which is a combination of channel shuffle and densely connected convolutional networks. Channel Shuffle is a technique used in CNN that aims to improve cross-channel correlation of features and network performance while maintaining computational efficiency by redistributing channels between different convolutional layers. Densely connected convolutional network is a network architecture where each layer is directly connected to the other layers of its own network. And each layer is directly connected to the other layers of its own network before it. This densely connected structure facilitates more efficient transfer of information and gradients and can reduce the number of parameters in the model. SDN is composed of Shuffle Dense Block (SDB) and the basic structure of SDB is shown in Fig. 3.

Figure 3 shows the basic structure of SDB. It is used to enhance channel coding performance in communication systems, mainly composed of input buffer, aliaser, and output buffer. Aliaser is a core part of SDB, mainly responsible for rearranging input data blocks to increase the spacing and correlation between data, and provide better error correction performance. Aliaser usually adopts different aliasing modes or algorithms, such as block aliasing, bit aliasing, interleaver, etc. The input cache is used to temporarily store input data blocks and wait for aliaser processing. The size and structure of the input cache usually match the working mode and requirements of aliaser. The output cache is used to store the data blocks output by the aliaser. The size of the output cache usually matches the size of the input cache to ensure the correctness and integrity of data during the aliasing process. After inputting the image into it, SDB will perform channel segmentation on the feature map, dividing it into two equal parts. After different feature extraction, the two feature maps are finally merged.

**Figure 4:**
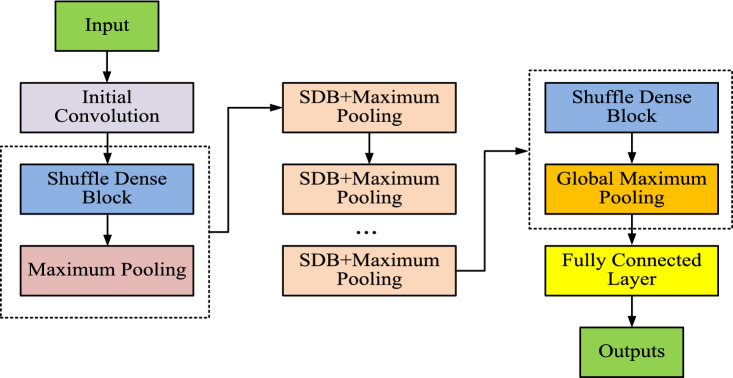
Flow chart of SDN operation.

In order to avoid feature disappearance and insufficient feature transfer during the feature transfer process, the study used multiple SDBs and channel aliasing technology to build the final SDN for identifying the grayscale image of the perceptual energy observation matrix^23^. The operation flow chart of SDN is shown in Fig. 4.

Figure 4 shows the operation flowchart of SDN, which shows that the final constructed SDN is composed of multiple SDB modules connected in series. In addition, Fig. 4 shows the operation flow of DN in detail. It is mainly divided into spectrum data input, initialization processing, convolution operation to extract signal features, pooling operation to reduce the size of the feature map to remove redundant information, global maximum pooling for feature dimensionalization, fully-connected layer for feature classification, and outputting the determination results of spectrum occupancy. Firstly, spectrum data are input. Then the data are initialized and convolution operation is performed. Next, a maximum pooling layer with a dimension of 2*2 and a step size of 2 is added behind each SDB module. It is used to reduce the size of the feature maps in the channel and remove redundancy and improve the computational speed of the model and avoid overfitting of the model training. Then a global maximum pooling layer is added behind the last SDB module, whose purpose is to fit the output features according to the number of channels. So the dimensionality reduction of the output features can be realized and the classification of the features can be facilitated by the final fully connected layer^24^. Finally, the features after the fully connected layer are categorized and output.

### Communication spectrum state prediction model design based on convolutional gated RNN

**Figure 5:**
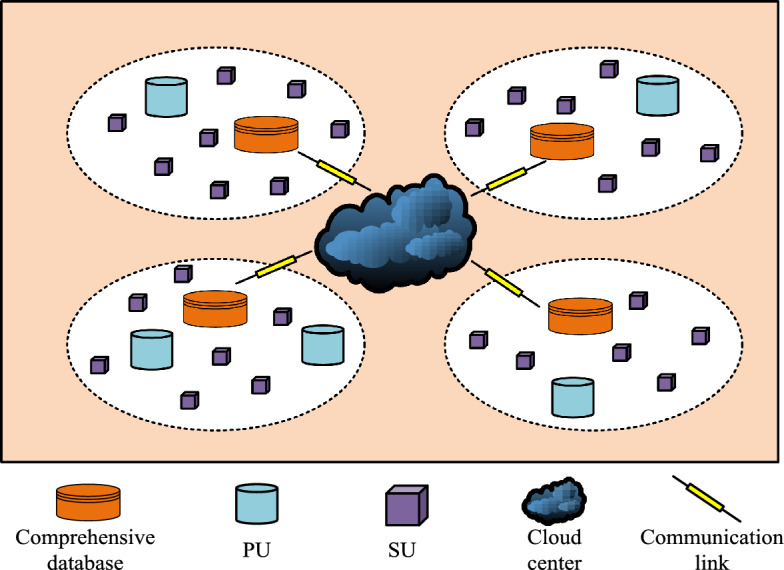
Spatial distribution map of the spectrum prediction model.

Spectrum prediction in CR scenarios can predict spectrum occupancy in advance and improve spectrum reuse efficiency^25,26^. Since spectrum occupancy has a strong correlation in time, deep neural network models based on extracting time-series spectrum features are widely used. However, most current models are limited to two-dimensional features in the frequency domain and time domain, ignoring the spatial domain features, or only single-step prediction, and the prediction results have certain limitations. This chapter proposes a prediction scheme based on the Convolutional Gated Recurrent Unit (ConvGRU) network, which combines three-dimensional features of time, space, and frequency for multi-slot long-term spectrum prediction, aiming to improve the prediction performance of the current spectrum prediction model. The spatial distribution structure of the spectrum prediction system is shown in Fig. 5.

The spatial distribution of the spectrum prediction system model is shown in Fig. 5. The whole space contains PUs, SUs, databases, cloud processing centers, and communication links, which are represented by different graphics. PUs and SUs are randomly distributed in the whole space. The whole space is divided into several small parts and a spectrum information database is deployed in each part. Finally, the cloud center is used to connect the paWhen the sensing task arrives, SU is able to perform spectrum sensing, which can be represented by Eqs. (6) and (7). Firstly, the value of the signal received by the SU participating in the sensing task is calculated as shown in Eq. (6).

6 $$y_{ij} \left( n \right) = \left\{ {\begin{array}{*{20}l} {\delta_{i} \left( n \right)} \\ {x_{ij} \left( n \right) + \delta_{i} \left( n \right)} \\ \end{array} \begin{array}{*{20}c} {} \\ {} \\ \end{array} \begin{array}{*{20}l} {H_{0} } \\ {H_{1} } \\ \end{array} } \right.$$

where $y_{ij} \left( n \right)$ represents the signal received by the $i$-th SU participating in the perception task $j$, $\delta_{i} \left( n \right)$ represents a mean of 0, $x_{ij} \left( n \right)$ represents the useful signal received by the $i$-th SU participating in the perception task $j$, and $H_{0}$ and $H_{1}$ respectively indicate that the spectrum is in an idle state and in use state.

After calculating $y_{ij} \left( n \right)$, the equation for $x_{ij} \left( n \right)$ is then obtained as shown in Eq. (7).

7 $$x_{ij} \left( n \right) = s_{j} \left( n \right)h_{ij} \left( n \right)$$

where $s_{j} \left( n \right)$ represents the transmission signal of the $j$-th PU and $h_{ij} \left( n \right)$ represents the channel coefficient between the $i$-th SU and the $j$-th PU.

SU not only performs the sensing task by performing energy superposition with multiple signal samples, but also uses these samples to identify false occupancy situations. By analyzing the statistical properties of the energy superposition values, it is possible to distinguish between real spectrum occupancy and pseudo-occupancy signals caused by environmental noise or other non-occupancy sources. The number of times each SU samples the signal from the PU during the perception time slot is denoted as $S$. The SU performs energy superposition by multiple signal sampling, which is calculated as shown in Eq. (8).

8 $$E_{ij} = \sum\limits_{n = 1}^{S} {\left| {y_{ij} \left( n \right)} \right|}^{2}$$

where $E_{ij}$ represents the energy superposition value.

**Figure 6:**
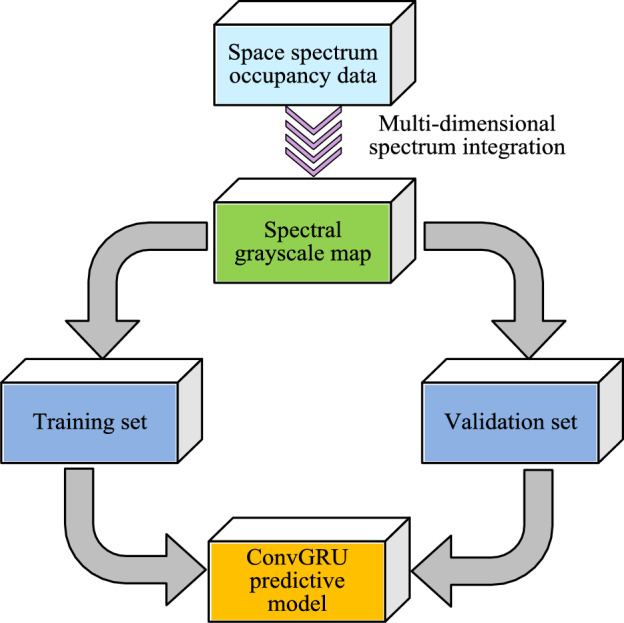
Flow chart of spectrum prediction.

Due to the distance between SU and PU in practical applications, it is necessary to set a decision gate to verify their status. The sensed data will be sent to the cloud center in the form of time-series spectrum between different communities. Then data integration will be carried out by the cloud center^27,28^. By filling in the matrix block, the specific situation of the temporal spectrum occupying the image can be obtained. Then, ConvGRU is used for spectrum prediction, and spectrum sensing tasks and spectrum resources are allocated based on the prediction results. The operation flow of the spectrum prediction algorithm is shown in Fig. 6.

**Figure 7:**
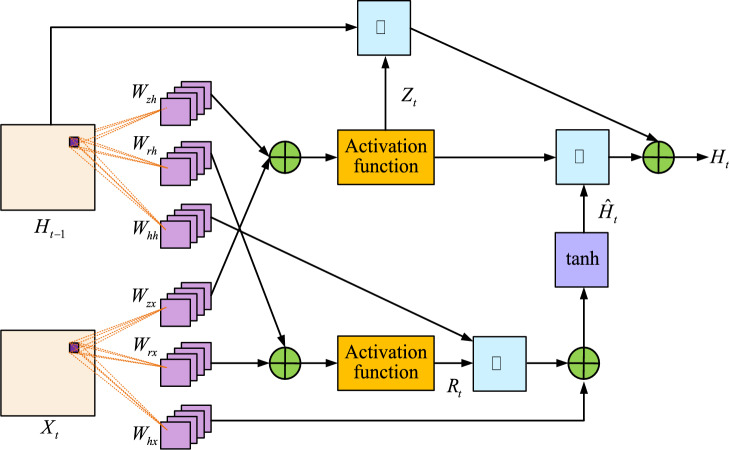
Feature extraction unit plot of the ConvGRU prediction model.

The operation flow chart of the spectrum prediction algorithm is shown in Fig. 6. From Fig. 6, the whole spectrum prediction can be divided into two parts: data preprocessing and model building. It exhaustively depicts the complete operation flow of the ConvGRU prediction model, from data preprocessing to the output of final prediction results. In the data preprocessing stage, the spatial spectrum occupancy data are first integrated multidimensionally to generate the time-series spectrum grayscale map. Next, when building the model, the preprocessed data are analyzed using ConvGRU. The model places special emphasis on the extraction of spatio-temporal features, and each ConvGRU unit is able to integrate the information of current and historical frames. Eventually, the model combines all the extracted features for prediction and outputs the future state of spectrum occupancy. By integrating multidimensional spectral features, not only can the input features of the network be increased, but also the prediction accuracy of the ConvGRU prediction model can be further improved. The feature extraction process of ConvGRU prediction model is shown in Fig. 7.

Figure 7 shows the feature extraction unit diagram of the ConvGRU prediction model. The ConvGRU prediction model constructed mainly includes two parts: spatial feature extraction and temporal feature extraction. When each frame of image is input into the network, it is first necessary to extract the spatial features of each input image through the built-in convolutional kernel in the ConvGRU unit. Secondly, the extracted spatial features are transmitted over time and used as input for the next ConvGRU unit. Finally, the output content of each unit in ConvGRU includes both the input features of the current unit and the output features of the historical unit^29^.

The specific extraction steps of spatial features are shown in Eqs. (9) to (12). Firstly, the value of ConvGRU update gate is calculated as shown in Eq. (9).

9 $$R_{t} = \sigma \left( {W_{rh} * H_{t - 1} + W_{rx} * X_{t} + b} \right)$$

where $X_{t}$ is the input image of the current unit, $H_{t - 1}$ represents the output feature of the previous unit, $R_{t}$ represents the reset gate of ConvGRU, $W_{rh}$ represents the convolutional kernel that extracts the output features of the previous unit from the reset gate, $W_{rx}$ represents the convolutional kernel that extracts the input features of the current unit from the reset gate, $b$ represents the offset of the current convolutional layer, $\sigma$ stands for activation function, and $*$ represents the convolution operation.

The formula for calculating the ConvGRU update gate output value is shown in Eq. (10).

10 $$Z_{t} = \sigma \left( {W_{zh} * H_{t - 1} + W_{zx} * X_{t} + b} \right)$$

where $Z_{t}$ represents the update gate output, $W_{zh}$ represents the convolutional kernel used by the update gate to extract historical output features, and $W_{zx}$ represents the convolutional kernel used by the update gate to extract the current input feature.

The sigmoid function is selected as activation function, and this activation function is used to set the reset gate output between [0, 1]. When the reset gate approaches 0, it indicates that the historical output features have a smaller impact on the hidden state of the current unit, which is more conducive to long-term prediction^30^. On the contrary, the reset gate approaches 1 closer, the short-term expectations are more favorable. The implicit state of the current unit is set to $\hat{H}_{t}$ in Eq. (11).

11 $$\hat{H}_{t} = \tanh \left( {R_{t} \circ \left( {W_{hh} * H_{t - 1} } \right) + W_{hx} * X_{t} + b} \right)$$

where $W_{hh}$ represents the convolutional kernel for extracting historical output features in the hidden state, $W_{hx}$ represents the convolutional kernel that extracts the current input feature in the hidden state, $\tanh$ is the hyperbolic tangent activation function, and $\circ$ is the Hadamard product.

The final output values of the spatial features in the ConvGRU network are shown in Eq. (12).

12 $$H_{t} = Z_{t} \circ \hat{H}_{t} + \left( {1 - Z_{t} } \right) \circ H_{t - 1}$$

Equation (12) shows the calculation formula for the current unit output.

**Figure 8:**
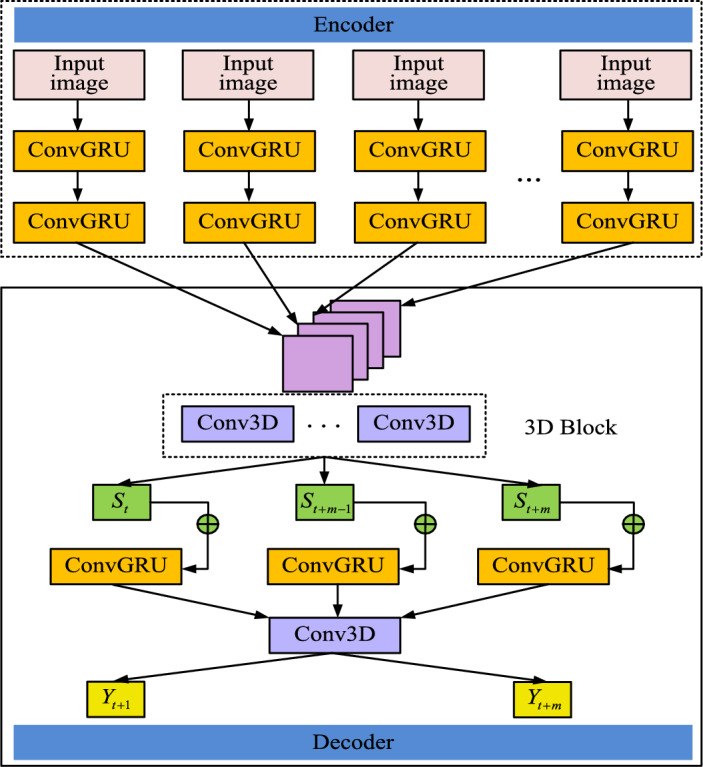
ConvGRU predicted network structure diagram.

The specific output value of the current unit can be calculated through Eqs. (9) to (12). Figure 8 shows the final designed ConvGRU prediction network structure.

The structure of the final designed ConvGRU prediction network is shown in Fig. 8. From Fig. 8, the entire spectrum prediction network structure mainly consists of an encoder and a decoder. In the encoder layer, it consists of two layers of ConvGRU units, and the output of each ConvGRU unit contains the input features of all previous frames. In the decoder layer, to avoid the excessive prediction error, the input sequence $\left\{ {H_{t - n} , \cdots ,H_{t} } \right\}$ is used as the input to the decoder, and then all the input features are integrated through the 3D Block layer.

The 3D Block layer contains several Conv3D sub-modules that not only extract features in the time dimension, but also capture subtle changes in channel conditions. Changes in channel conditions, such as signal fading and interference, directly affect the reliability of the signal and the accuracy of spectrum perception. Therefore, the constructed model is able to predict spectrum occupancy more accurately and improve the success rate of spectrum sensing by analyzing these temporal features. The formula for feature extraction using 3D Block layer is shown in Eq. (13).

13 $$S_{t} = g\left( {W_{st} * \left[ {H_{t - n} ,H_{t - n + 1} , \cdots ,H_{t} } \right]} \right)$$

where $W_{st}$ represents the convolutional kernel used by convolutional 3D submodule in 3D block that outputs $S_{t}$ to extract features and $g\left( \cdot \right)$ is activation function.

The most effective prediction information can be extracted by integrating all the feature information, which is used as the input to the prediction layer of the decoder to obtain the final prediction result. The formula for the final prediction result is shown in Eq. (14).

14 $$Y_{t + 1} = cg\left( {\left( {S_{t} + H_{t} } \right),H_{t} } \right)$$

where $Y_{t + 1}$ represents the prediction result of $t + 1$ frames and $cg\left( \cdot \right)$ represents the nonlinear transformation of ConvGRU units.

## Performance analysis and application of communication collaborative spectrum perception model and prediction model

To further verify the performance of the perception model and the prediction model, this study used multiple indicators such as network iteration performance, perception accuracy, prediction accuracy, and prediction time to validate the above models. In addition, the study also selected common perception models and prediction models for comparison, with the aim of further proving the proposed model’s effectiveness. These final results confirm that the proposed spectrum sensing model and prediction model have good performance and can efficiently and successfully perceive spectrum information and predict SUbsequent trend of spectrum changes.

### Performance analysis of communication collaborative spectrum perception model based on SDN

To further test the performance of the spectrum-aware model, the study first builds the running environment of the algorithm using Python programming language and Tensorflow framework. Then the basic parameters of the SDN network were set with reference to the Densely Connected Convolutional Networks-121 (Dense Net-121), and its network growth rate was set to 32.

To ensure the validity and accuracy of this study, historical spectrum occupancy data from a major wireless communication operator in China are used as the experimental dataset. The dataset covers the spectrum utilization of a large city over the past year, including spectrum occupancy information for different time periods and different frequency bands. The total records of the dataset are about 1 million, spanning from January 2022 to December 2022, and the frequency covers the wireless spectrum from 2.4 to 5 GHz. Data preprocessing consisted of thoroughly cleaning the data to remove incomplete or erroneous records, and then normalizing the data to ensure that all values lie between 0 and 1. In addition, this study extracted key features such as signal strength and channel occupancy from the raw data. These are the basis for model training. The entire dataset contains about 1 million records, which is sufficient to ensure that the model can fully learn the complexity and diversity of spectrum data. To effectively evaluate the performance of the proposed model, the dataset is partitioned into a training set, a validation set, and a test set. Among them, 70% of the data is used as the training set to construct and optimize the model. 15% of the data is used as the validation set to adjust the model parameter. The remaining 15% is used as the test set to evaluate the generalization ability and prediction accuracy of the model. The proportions of the dataset are determined after considering the optimal training and evaluation of the model. 70% of the training set is sufficient to ensure that the model is able to adequately learn and adapt to the characteristics of the spectral data, whereas the 15% validation set allows for effective tuning and optimization of the model parameters to prevent overfitting. The remaining 15% test set provides a sufficient amount of data to evaluate the model's generalization ability and prediction accuracy on unseen data. This split ratio is determined after several experiments after weighing data utilization and model evaluation accuracy to ensure optimal model performance in all aspects.

**Figure 9:**
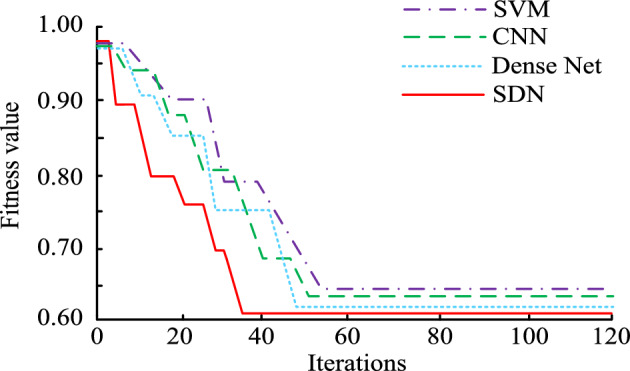
Iterations of different perceptual models.

In this study, the proposed method was simulated using Support Vector Machine (SVM), CNN, SDN and Densely Connected Convolutional Networks (Dense Net), respectively, without changing the actual architectures of the four models. All the models were tested for performance under the same experimental conditions. The study first compared the iteration of different perception models in Fig. 9.

Figure 9 shows the iterative curves of four perception models. In Fig. 9, as the iteration increases, the optimal fitness values of the four perception models, SVM, CNN, Dense Net, and SDN, all decrease accordingly. When the iteration is 36, SDN can first iterate to a stable state, and the optimal fitness value of SDN is 0.61. When the iteration is 47, Dense Net can iterate to a stable state, and the optimal fitness value of the model is 0.63. Compared to SDN and Dense Net, the iterative state of SVM and CNN is more unstable, requiring 55 and 50 iterations, respectively, to reach a stable state. The optimal fitness values for SVM and CNN at steady-state are 0.65 and 0.64, respectively.

**Figure 10:**
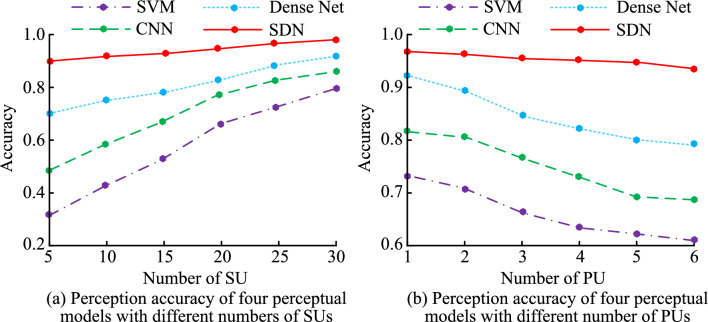
Perception accuracy of the four models with different SU and PU numbers.

Figure 10 shows the perceptual accuracy of four models under different numbers of SU and PU. Figure 10a shows the perceptual accuracy of four models under different SU numbers. According to Fig. 10a, as SU increases, the perceptual accuracy of all four models shows an upward trend. Compared to the three models of SVM, CNN, and Dense Net, the perceptual accuracy of SDN is not affected by SU and can always maintain above 0.9, with a maximum of 0.99. The perceptual accuracy of SVM, CNN, and Dense Net continues to improve with SU increasing. Finally, the highest perceptual accuracy values of SVM, CNN, and Dense Net were 0.77, 0.85, and 0.91, respectively. Figure 10b shows the perceptual accuracy of four models under different numbers of PU. According to Fig. 10b, as PU increases, the perceptual accuracy of the four models shows a continuous decreasing trend. When PU is 1, the four models SVM, CNN, Dense Net, and SDN have the highest perceptual accuracy, which are 0.73, 0.82, 0.92, and 0.97, respectively.

**Figure 11:**
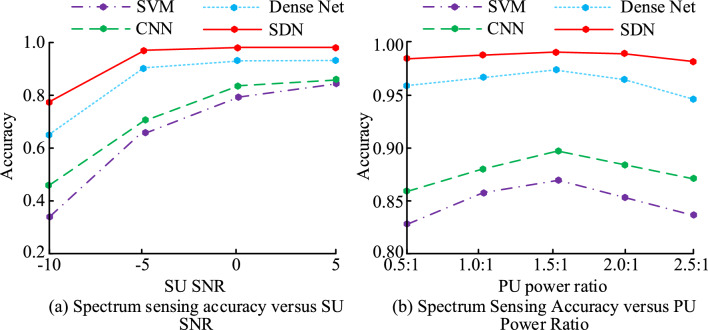
Results for the influence of SU SNR and PU power ratio on the accuracy of the perceptual model.

Figure 11 shows the results of the influence of SU SNR and PU power ratio on the perception model’s accuracy. Figure 11a shows the impact of different SU SNRs on the perceptual accuracy of these four models. According to Fig. 11a, as SU SNR continues to increase, the perceptual accuracy of all four models shows an upward trend. Compared to SVM and CNN models, Dense Net and SDN models have higher perceptual accuracy. Figure 11b shows the impact of different PU power ratios on the perception accuracy of the four models. According to Fig. 11b, as PU power ratio increases, the perceptual accuracy of these four models shows a trend of first increasing and then decreasing. When the PU power ratio is 1.5:1, SVM, CNN, Dense Net, and SDN models have the highest perceptual accuracy, with values of 0.862, 0.891, 0.964, and 0.983, respectively.

Table 1 shows the accuracy of the four perception models under specific SU SNR and PU power ratios. According to Table 1, when SU SNR increases from − 10 to 5, the perceptual accuracy of all four models increases to varying degrees. When SU SNR is − 10, − 5, 0, and 5, the perceptual accuracy of SDN model is 0.795, 0.977, 0.980, and 0.982, respectively. When PU power ratio increases from 0.5:1 to 2.5:1, the perceptual accuracy of all four models shows a trend of first increasing and then decreasing. When PU power is 0.5:1, 1.0:1, 1.5:1, 2.0:1, and 2.5:1, the perceptual accuracy of SDN model is 0.962, 0.975, 0.983, 0.981, and 0.960, respectively.

**Table 1:** Accuracy values of the perceptual models under different SU SNR and PU power ratios.

Model	SU SNR				PU power rate				
	− 10	− 5	0	5	0.5:1	1.0:1	1.5:1	2.0:1	2.5:1
SVM	0.382	0.643	0.775	0.818	0.828	0.855	0.862	0.848	0.836
CNN	0.468	0.712	0.820	0.835	0.857	0.874	0.891	0.882	0.860
Dense Net	0.634	0.889	0.901	0.905	0.959	0.962	0.964	0.960	0.948
SDN	0.795	0.977	0.980	0.982	0.962	0.975	0.983	0.981	0.960

### Performance analysis of communication collaboration SPM based on ConvGRU

**Figure 12:**
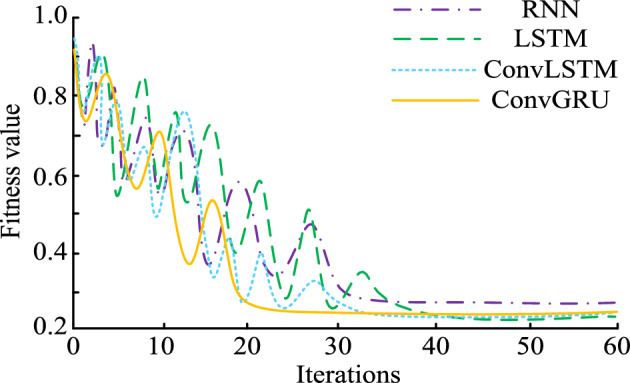
The iterations of different prediction models.

In addition to testing the performance of the spectrum sensing model, the study further tests the performance of the spectrum prediction model. The experimental environment setup and dataset in "Design of spectrum sensing model for communication cooperation based on channel aliasing dense connection network" section are still used for model performance testing. The proposed ConvGRU consists of two convolutional layers, each with 64 convolutional kernels of 3 × 3 size with a step size of 1 and no padding. In the pooling layer, the pooling window is 2 × 2. The pooling layer is followed by GRU layers, each with 128 neurons. To prevent overfitting, the network was trained using an Adam optimizer using a dropout rate of 0.5 after each fully connected layer and choosing the mean square error as the loss function. The initial learning rate was set to 0.001, the batch size was 32, and a total of 50 cycles were trained. Firstly, the iterative training performance of four prediction models was compared in Fig. 12.

Figure 12 shows the network iteration curves for four types of SPMs. From Fig. 12, as the number of iterations increases, RNN, LSTM, Convolutional Neural Networks-Long Short-Term Memory (ConvLSTM) and the optimal fitness values of the four spectrum prediction models of ConvGRU all decreased accordingly. Among them, ConvGRU can iterate to a stable state as quickly as possible. When the iteration is 20, this model begins to converge, and its optimal fitness value this time is 0.25. In addition, RNN, LSTM, and ConvLSTM models require 32, 44, and 35 iterations, respectively, to reach a stable state.

**Figure 13:**
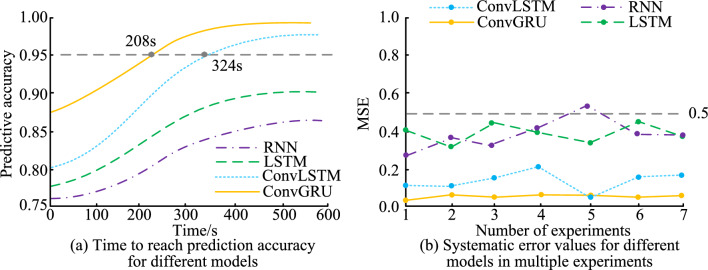
Prediction accuracy and systematic error performance of different models.

Figure 13 shows the time it takes for the four SPMs to achieve prediction accuracy values and the system error values of the four SPMs in multiple experiments. According to Fig. 13a, the highest prediction accuracy achievable by RNN and LSTM is 0.86 and 0.90, respectively. Compared to RNN and LSTM, ConvLSTM and ConvGRU can achieve higher prediction accuracy values. When ConvGRU reaches a predictive accuracy value of 0.95, the running time of this model is 208 s. This time required for ConvGRU to reach a predictive accuracy of 0.95 is calculated based on the size of the dataset and the processing power. This time frame is the average time obtained by iteratively training and testing the model on a specific hardware configuration. Specifically, this time is the average time taken for the model to reach 0.95 prediction accuracy from the start of training when processing a dataset containing millions of pieces of wireless spectrum data. Similarly, when ConvLSTM reaches the value of 0.95 prediction accuracy, the running time of this model is 324. Compared with the running time of ConvLSTM and ConvGRU, ConvGRU can achieve higher prediction accuracy values faster, so the model has better stability and higher prediction accuracy in the prediction process. According to Fig. 13b, compared to other three prediction models, the system error values of ConvGRU in multiple tests are all below 0.1 and far lower than other three models.

**Figure 14:**
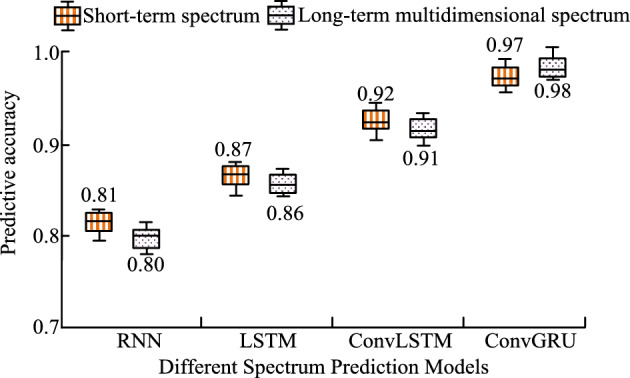
Different prediction models for the two times.

Figure 14 shows the prediction accuracy values of four different SPMs for short-term and long-term multidimensional spectra. According to Fig. 14, the prediction accuracy values of RNN for short-term and long-term multidimensional spectra are 0.81 and 0.80, respectively. The prediction accuracy values of LSTM for short-term and long-term multidimensional spectra are 0.87 and 0.86, respectively. The prediction accuracy values of ConvLSTM for short-term and long-term multidimensional spectra are 0.92 and 0.91, respectively. The prediction accuracy values of ConvGRU for short-term and long-term multidimensional spectra are 0.97 and 0.98, respectively. Overall, ConvGRU has better spectrum prediction performance.

Table 2 shows the prediction accuracy of ConvGRU and ConvLSTM models for short-term and long-term multidimensional spectra with time steps of 10–100. By comparing the predicted values of the two models, ConvGRU has higher accuracy in predicting short-term and long-term multidimensional spectra than ConvLSTM. In addition, as the time step increases, the spectral prediction accuracy of ConvGRU does not significantly change, while the prediction accuracy of ConvLSTM model will significantly decrease with time step increasing.

**Table 2:** Spectrum prediction accuracy values of the two models at different time steps.

Time step	ConvGRU		ConvLSTM	
	Short-term spectrum	Long-term multidimensional spectrum	Short-term spectrum	Long-term multidimensional spectrum
10	0.978	0.982	0.925	0.916
20	0.974	0.982	0.911	0.912
30	0.971	0.980	0.905	0.908
40	0.970	0.978	0.892	0.878
50	0.968	0.979	0.870	0.861
60	0.968	0.975	0.854	0.855
70	0.965	0.974	0.833	0.789
80	0.964	0.973	0.825	0.762
90	0.965	0.974	0.763	0.731
100	0.962	0.972	0.741	0.725

## Discussion

To effectively improve the utilization efficiency of spectrum resources, this study uses SDN and ConvGRU networks to build wireless communication spectrum sensing models and prediction models, respectively. Research results show that the perception accuracy of the proposed SDN perception model reaches 0.99 under different numbers of SUs, while the ConvGRU prediction model can achieve a prediction accuracy of 0.95 in only 208 s. The comparison of the results is shown below. First of all, regarding the novelty of the research, it combines the spatial feature extraction capabilities of CNN with the time-series processing advantages of gated recurrent unit to achieve high-precision and fast-response spectrum prediction. In the reference^31^, the system focuses on improving the spectrum efficiency of wireless communication systems, while this study shows more obvious advantages in terms of accuracy and response speed of spectrum data processing in dynamic environments. Secondly, in terms of handling complex long-term dependencies and dynamic changes, this study demonstrates higher flexibility and accuracy compared with the radar and massive Multiple Input Multiple Output (MIMO) cellular coexistence study in the reference^32^. Reference^32^ uses complex mathematical models to deal with wireless communication environments, but this study uses deep learning technology to show higher adaptability in predicting future spectrum usage. Furthermore, considering the complexity of real-world application scenarios, the stability and adaptability of the model used in this study under different user densities and signal interference are in sharp contrast to the communication spectrum prediction methods based on homography theory and Hidden Markov Model (HMM) proposed in the reference^33^. Although the reference^33^ addresses the challenge of prediction in specific application scenarios, this study provides broader applicability and reliability.

The main contributions of this study are as follows. The research successfully constructs a spectrum sensing model based on SDN. This model can accurately perceive the usage of spectrum resources according to different number of users, and the sensing accuracy can reach up to 0.99. In addition, a spectrum prediction model based on ConvGRU is also proposed in the study. The model can achieve a prediction accuracy of 0.95 in only 208 s, showing higher prediction accuracy and faster prediction speed than other spectrum prediction models. By combining the sensing and prediction models, this research provides a more efficient and accurate spectrum management solution for the radio communications field. This solution can help wireless communication systems better understand and predict the use of spectrum resources, thereby improving overall network efficiency and resource utilization.

In summary, this research has made significant contributions in the wireless communication spectrum management, especially in terms of the accuracy of spectrum sensing and the speed of spectrum prediction. Through these innovations, this research provides new perspectives and tools for spectrum resource optimization and management of wireless communication systems.

## Conclusion

To better perceive and predict changes in the communication spectrum, this study constructs a spectrum sensing model and SPM and tests the performance of the model. This can assist the communication system in making timely resource adjustments based on network conditions. For the spectrum sensing model, SDN reaches a stable state after 36 iterations and has a high perception accuracy of 0.61. In contrast, the iterative state of SVM and CNN models is relatively unstable, requiring 55 and 50 iterations, respectively, to reach a stable state. In terms of perceptual accuracy, SDN exhibits high stability under different numbers of SU, consistently maintaining above 0.9, with a maximum of 0.99. In contrast, the perceptual accuracy of SVM, CNN, and Dense Net models gradually improves with the increase of SU numbers, reaching the highest of 0.77, 0.85, and 0.91, respectively. Under different numbers of PUs, when the number of PUs is 1, SDN model exhibits the highest perceptual accuracy of 0.97, while SVM, CNN, and Dense Net models have perceptual accuracy of 0.73, 0.82, and 0.92, respectively. For SPM, compared to RNN, LSTM, and ConvLSTM models, ConvGRU can reach a stable state faster and has a lower optimal fitness value of 0.25 in iterative training. In terms of prediction accuracy and stability, the running time required for ConvGRU to reach a prediction accuracy value of 0.95 is 208 s, while the ConvLSTM model requires 324 s. ConvGRU has higher prediction accuracy and better stability compared to ConvLSTM model. Future research can further explore the performance of other models and algorithms and consider a wider range of practical application scenarios to achieve more accurate and reliable spectrum sensing and prediction.

## Data Availability

The datasets used and/or analysed during the current study available from the corresponding author on reasonable request.

## Abbreviations

LTE: Long-term evolution
5G: 5Th generation
CNN: Convolutional neural network
LSTM: Long short-term memory
EEG: Electroencephalography
SPM: Spectral prediction model
CNN-BiLSTM: Convolutional neural network-bidirectional long short-term memory
CR: Cognitive radio
RNN: Recurrent neural network
NOMA: Non-orthogonal multiple access
ConvLSTM: Convolutional neural networks-long short-term memory
HMM: Hidden Markov model
CR-NOMA: Cognitive radio-non-orthogonal multiple access
PU: Primary user
SU: Secondary user
SDN: Shuffle dense network
SDB: Shuffle dense block
ConvGRU: Convolutional gated recurrent unit
Dense Net-121: Densely connected convolutional networks-121
SVM: Support vector machine
Dense Net: Densely connected convolutional networks
SNR: Signal to noise ratio
MIMO: Multiple input multiple output
